# Calcium Sensing Receptors Mediate Local Inhibitory Reflexes Evoked by L-Phenylalanine in Guinea Pig Jejunum

**DOI:** 10.3389/fphys.2017.00991

**Published:** 2017-12-04

**Authors:** Rachel M. Gwynne, Kenny D. K. N. Ly, Laura J. Parry, Joel C. Bornstein

**Affiliations:** ^1^Department of Physiology, University of Melbourne, Parkville, VIC, Australia; ^2^School of BioSciences, University of Melbourne, Parkville, VIC, Australia

**Keywords:** extracellular CaSR, L-Phenylalanine, L-glutamate, inhibitory reflexes, ATP, 5-HT, AMPA receptors

## Abstract

Amino acids applied to the mucosa evoke inhibitory reflexes in guinea-pig jejunum, but the receptors involved in sensory transduction are still unclear. One promising candidate is the extracellular calcium sensing receptor (CaSR), which is expressed by mucosal enteroendocrine cells and is preferentially activated by aromatic L-amino acids. We tested this by applying various amino acids to the mucosa and recording the resulting inhibitory junction potentials (IJPs) in nearby circular smooth muscle via intracellular recording. The CaSR is stereospecific and L-Phenylalanine evoked a significantly larger response than D-Phenylalanine when both were applied to the same site. The same pattern was seen with L- and D-Tryptophan, another aromatic amino acid. The CaSR is preferentially activated by aromatic amino acids and responses to L-Leucine and L-Lysine were significantly lower than those to L-Phenylalanine applied to the same site. Responses to L-Phenylalanine were dose-dependently suppressed by blockade of the CaSR with NPS2143, a CaSR antagonist, and mimicked by mucosal application of cinacalcet, a CaSR agonist. Responses to cinacalcet had similar pharmacology to that of responses to L-Phenylalanine, in that each requires both P2 purinoreceptors and 5-HT receptors. L-Glutamate evoked IJPs similar to those produced by L-Phenylalanine and these were depressed by blockade of P2 receptors and 5-HT_3_ plus 5-HT_4_ receptors, but NPS2143 was ineffective. The AMPA receptor antagonists DNQX (10 μM) and CNQX (10 μM) reduced IJPs evoked by L-Glutamate by 88 and 79% respectively, but neither BAY367260 (mGluR5 antagonist) nor 2APV (NMDA antagonist) affected IJPs evoked by L-Glutamate. We conclude that local inhibitory reflexes evoked by aromatic L-amino acids in guinea pig jejunum involve activation of CaSRs which triggers release of ATP and 5-HT from the mucosa. L-Glutamate also evokes inhibitory reflexes, via a pathway that does not involve CaSRs. It is likely there are multiple receptors acting as sensory transducers for different luminal amino acids.

## Introduction

The primary functions of the gastrointestinal tract are controlled by the enteric nervous system (ENS) but the mechanisms by which luminal nutrients are coupled to appropriate changes in intestinal motility remain poorly understood. In recent years, much research has focused on identifying the sensory molecules involved in the detection of different types of nutrients. There is strong evidence to suggest that at least one way this detection process or “chemosensation” occurs is via activation of specialized receptors for nutrients on mucosal enteroendocrine (EE cells) or other types of epithelial cells which release their contents (e.g., 5-HT, CCK) basally to excite intrinsic (and extrinsic) sensory nerve terminals lying in the lamina propria (Sternini et al., 2008; Wellendorph et al., 2009; Hong et al., 2012). Activation of these neurons in turn excites enteric neural circuits responsible for generating motor patterns. There is now abundant evidence that some EE cells express surface receptors that bind particular nutrients (Dyer et al., 2005; Guan et al., 2006; Karaki et al., 2006). Receptors for short and long chain fatty acids (FFARs) and receptors and molecules involved in glucose and sugar sensing (sweet taste T1R2/T1R3 receptor and alpha-gustducin) have been localized to mucosal EE cells in several species and in some EE cell lines (Rozengurt and Sternini, 2007; Bertrand, 2009; Wellendorph et al., 2009). In addition, the T1R1/T1R3 heterodimer which senses the distinct taste of “umami” elicited by L-Glutamate (L-Glu), and the metabotropic glutamate receptor mGluR_4_, have both been shown to function as broad sensors of L-amino-acids (Nelson et al., 2002; Chaudhari et al., 2009).

We have previously demonstrated that luminal fatty acids (Gwynne et al., 2004b) or the amino acids, L-Phenylalanine (L-Phe) or L-Tryptophan (L-Trypt) evoke neurally mediated segmentation in the guinea pig small intestine *in vitro* (Gwynne et al., 2004a). Mucosal application of these same L-amino acids evokes neural reflexes leading to local inhibitory responses in the circular muscle. The latter involves endogenous release of 5-HT, ATP, and probably another mediator from the mucosa, but not cholecystokinin (CCK) (Gwynne and Bornstein, 2007). This suggests EE cells are involved in sensing L-amino acids in the guinea-pig small intestine. Recent studies suggest one possible sensory receptor responsible for detecting aromatic amino acids, and L-Phe in particular, is the extracellular CaSR. The CaSR is primarily responsible for detecting extracellular Ca^2+^ but is also known to be allosterically activated by aromatic L-amino acids such as L-Phe and L-Trypt, i.e., not activated by the corresponding D-isomers (Conigrave et al., 2000). Recent studies have identified CaSRs on isolated mouse intestinal I cells (containing CCK) and have demonstrated CaSR activation by L-Phe (and L-Trypt) resulting in CCK release (Liou et al., 2011; Wang et al., 2011). However, the physiological coupling of L-amino acid sensing by CaSRs to the activation of enteric neural reflex pathways and neural circuits involved in generating nutrient induced motility changes has not been demonstrated. Further, it is not yet clear if CaSRs are involved in the release of other sensory mediators such as 5-HT or ATP from different types of EE cells.

In this study we used intracellular recording from circular smooth muscle and local mucosal application of aromatic and non-aromatic amino acids together with the CaSR agonist, cinacalcet, and antagonist NPS2143, to investigate whether the extracellular CaSR is involved in sensing amino acids in guinea pig jejunal mucosa leading to the activation of inhibitory neural reflex pathways. We also investigated whether reflexes evoked by mucosal application of L-Glu involved similar mechanisms to those activated by the aromatic amino acids.

## Materials and methods

### Electrophysiology

Guinea-pigs (200–380 g) of either sex were sacrificed in accordance with the National Health and Medical Research Council (Australia) guidelines, and approved by the University of Melbourne Animal Experimentation Ethics Committee. Segments of jejunum (5 cm in length) were removed, flushed clean, and placed in oxygenated (95% O_2_, 5% CO_2_) physiological saline (composition in mM: NaCl 118, KCl 4.6, CaCl_2_ 2.5, MgSO_4_ 1.2, NaH_2_PO_4_ 1, NaHCO_3_ 25, d-glucose 11) containing hyoscine (1 μM) to minimize muscle contractions during intracellular recordings. The segments were dissected to allow access to the circular muscle (CM) on one half of the preparation leaving the mucosa intact on the circumferentially adjacent half, as described previously (Gwynne and Bornstein, 2009). The preparation was left to equilibrate for 1–2 h before commencing the experiment.

CM recordings were made next to intact mucosa circumferentially opposite and slightly oral to the recording area using conventional intracellular recording techniques (Gwynne and Bornstein, 2007). Inhibitory junction potentials (IJPs) recorded in the CM in response to chemical and electrical stimuli applied to the mucosa were examined.

### Chemical stimulation of the mucosa

Solutions of L- and D-amino acids (all 30 mM) were made up daily in physiological saline. Previous studies have shown that concentrations of L-Phe greater than 20 mM are required for CCK release via activation of CaSRs (Hira et al., 2008; Liou et al., 2011). Furthermore, we have previously reported that L- amino acid concentrations ranging from 1 to 30 mM evoke segmenting behavior in motility experiments (Gwynne et al., 2004a). L-amino acids were transiently applied to the mucosa (duration 150 ms) and tested at several locations to identify sites that produced the largest consistent IJPs. Four to five trials (2–3 min apart) with each L-amino acid were recorded before the corresponding D-isomer was applied at the same site. The L-amino acid was then retested to control for rundown of the response. A similar protocol was used to test the effects of antagonists. A minimum of 4–5 responses were recorded before antagonists were added to the bath solution. 4-5 responses were recorded with antagonists present (10–20 min) before the drugs were washed from the bath (20–30 min). Further responses were recorded after the washout period to determine if any effects seen in the presence of antagonists were reversible. Stock solutions of antagonists were initially made up in distilled water and diluted to working concentrations on the day of the experiment. The mean latencies and amplitudes of IJPs evoked by chemical stimuli were calculated. Volume and vehicle controls have been performed previously (Gwynne and Bornstein, 2007).

### Electrical stimulation

A unipolar stimulating electrode (50 μm stainless steel insulated with 15 μm Teflon) was used to deliver single pulses (1–2 mA, duration 0.5 ms, Master-8 stimulator, ISO-flex stimulus isolation unit, AMPI, Jerusalem, Israel) to the mucosa close to the location where chemical stimuli were applied. This was to test whether an intact neural pathway was present from that part of the mucosa to the recording site. Also, a comparison between the effects of antagonists on chemical vs. electrical stimuli was used to help identify the location(s) of receptors involved in the reflex pathway(s) activated. The mean latencies and amplitudes of IJPs evoked by electrical stimuli were calculated. Controls for stimulus spread have been conducted previously (Gwynne and Bornstein, 2007).

### Drugs

Drugs used in these experiments included; hyoscine, pyridoxal phosphate-6-axophenyl-2′-4′-disulfonic acid (PPADS), L-Alanine, L-Phenylalanine, L-Tryptophan, L-Lysine, L-Leucine, L-Glutamate, D-Alanine, D-Tryptophan, D-Phenylalanine, 6,7-dinitroquinoxaline-2,3-dione (DNQX), 6-cyano-7-nitroquinoxaline-2,3-dione (CNQX), DL-2-amino-5-phosphonopentanoic acid (2AP5), inosine monophosphate (IMP) (all from Sigma Aldrich NSW, Australia), tetrodotoxin (TTX, Alomone Labs, Israel), tropisetron (Sandoz Pharma, Switzerland), BAY367260 (Tocris, USA) cinacalcet, and NPS2143 (kind gifts from Professor Arthur Christopolous, Department of Pharmacology, Monash University, VIC, Australia).

### Analysis and statistics

Data are presented as mean ± SEM unless otherwise stated. Statistical comparisons were made using paired and unpaired *t*-tests and repeated measures ANOVA where appropriate. *P*-values < 0.05 were taken to indicate statistical significance.

## Results

Baseline CM activity consisted primarily of spontaneous IJPs and membrane potential depolarizations that sometimes triggered action potentials. When CM impalements were lost as a result of muscle contractions, the CM was re-impaled as close as possible to the original location taking advantage of the syncytial nature of the smooth muscle. Circular muscle cells with resting membrane potentials (RMP) between −45 and −65 mV were studied.

Throughout this study L-Phe was used as the basic stimulus with the effects of other amino acids, agonists, and antagonists compared to this aromatic amino acid.

### Structure-activity relationship for CaSR

**Figure 1:**
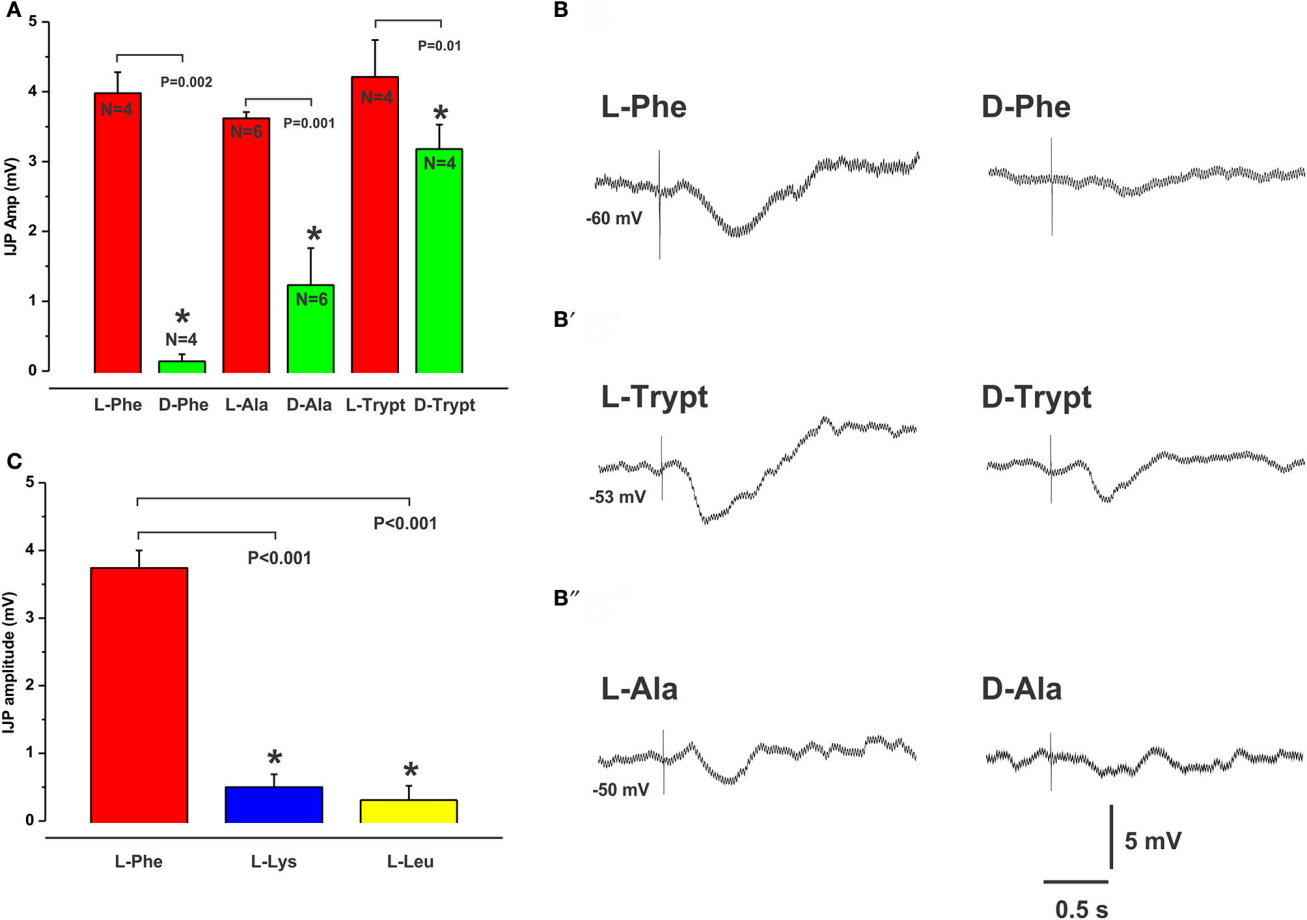
Stereospecificity of inhibitory responses evoked by L- and D-amino acids match the activation profile of the CaSR. The histogram in **(A)** shows the mean amplitudes of IJPs evoked by mucosal application of L-Phe, L-Ala, and L-Trypt compared with their corresponding D-isomers (each 30 mM). In each case the D-isomer evoked significantly smaller responses when tested at the same location as the L-isomer. The CaSR is known to be activated by L- but not by D- amino acids. **(B,B′,B″)** show intracellular recordings of IJPs evoked in circular muscle cells in response to the L- (left panel) and D- (right panel) amino acids. The histogram in **(C)** shows the mean amplitudes of IJPs evoked by L-Lys and L-Leu which were significantly smaller than those evoked by L-Phe. L-Lys and L-Leu are known to be less potent activators of the CasR. In all histograms to follow, mean IJP amplitudes (mV) are represented on the Y axis and “^*^” denotes significance at *P* < 0.05 compared with control (*P*-values and Ns are shown).

The extracellular CaSR is activated by the L-isomers of aromatic amino acids. We confirmed our previously published finding (Gwynne and Bornstein, 2007) that L-Phe applied to specific “hot-spots” on the mucosa evoked IJPs in neighboring circular smooth muscle cells. Responses consisted of IJPs (amplitude range 3–10 mV, latency range 150–300 ms), which were sometimes followed by a slow depolarization as reported previously (Gwynne and Bornstein, 2007). The inhibitory responses were the focus of the present study. These responses were mimicked by local application of another aromatic amino acid L-Trypt, and by application of L-Alanine (L-Ala), a branched chain amino acid that is moderately potent at CaSRs (Wellendorph et al., 2009). In contrast, the D-isomer of phenylalanine evoked only 1 IJP in 4 experiments when tested at the same site as the L-isomer (L-Phe mean IJP amp 4.0 ± 0.3 mV, D-Phe 0.1 ± 0.1 mV, *N* = 4, *P* = 0.002, Figures 1A,B). Responses to tryptophan and alanine were also found to be stereospecific (L-Trypt mean amp 4.2 ± 0.5 mV, D-Trypt 3.2 ± 0.4 mV, *N* = 4, *P* = 0.01, Figures 1A,B′; L-ala 3.6 ± 0.1 mV, D-ala 1.2 ± 0.5 mV, *N* = 6, *P* = 0.001, Figures 1A,B′′). There were no significant differences in the latencies of L-amino acid evoked responses compared with D-amino acid responses (mean latencies: L-Phe 0.18 ± 0.01 s, D-Phe 0.25 s, *N* = 1; L-Trypt 0.21 ± 0.02, D-Trypt 0.21 ± 0.01 s, *N* = 4; L-Ala 0.20 ± 0.02 s, D-Ala 0.23 ± 0.02, *N* = 4).

IJPs evoked by the non-aromatic amino acids L-Leucine (L-Leu) and L-Lysine, (L-Lys), which are less potent activators of the CaSR, had smaller amplitudes and were evoked less often when tested at sites that consistently produced IJPs evoked by L-Phe (mean IJP amp L-Phe 3.7 ± 0.3 mV, L-Lys 0.5 ± 0.2 mV, L-Leu 0.3 ± 0.2 mV, both *N* = 8 and *P* < 0.001, Figure 1C). When recorded, the latencies of IJPs evoked by L-Leu (mean latency: 0.24 ± 0.03 s, *N* = 3) and L-Lys (0.21 ± 0.01 s, *N* = 5) did not differ significantly from L-Phe responses (0.19 ± 0.01 s, *N* = 8).

### CaSRs are involved in L-AA evoked IJPs

**Figure 2:**
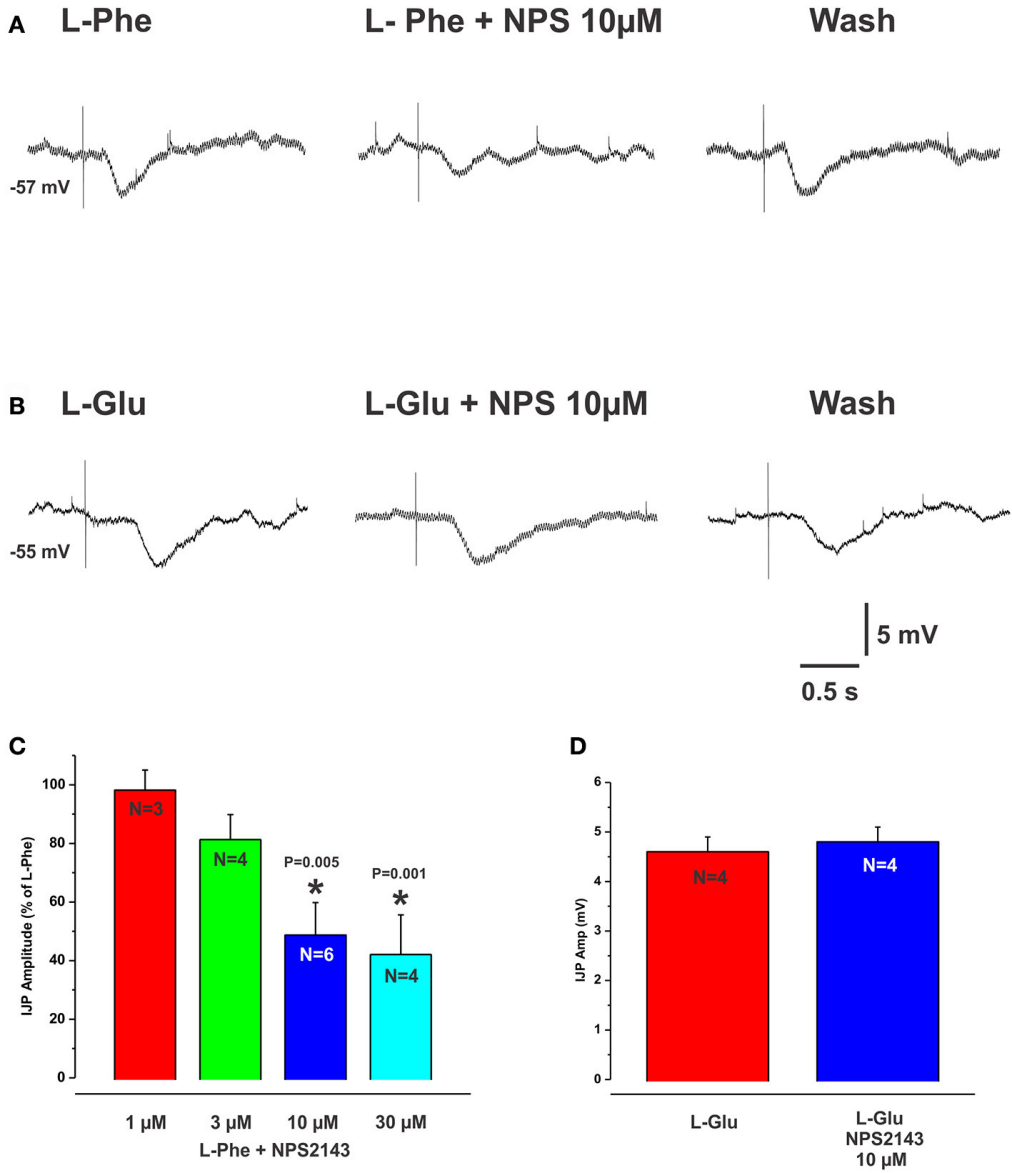
The CaSR antagonist NPS2143 reduced IJPs evoked by L-Phe but not those evoked by L-Glu. **(A,B)** (left panels) show intracellular recordings of IJPs evoked by L-Phe and L-Glu. The CaSR antagonist NPS2143 (10 μM) when added to the bathing solution, reversibly reduced IJPs evoked by L-Phe (**A**, middle panel, histogram in **C**) but IJPs evoked by L-Glu were unaffected (**B**, middle panel, histogram in **D**). The histogram in **(C)** shows the effects of various concentrations of NPS2143 (bath applied) on the amplitudes of L-Phe evoked IJPs. NPS 2143 reversibly reduced L-Phe evoked IJPs at 10 and 30 μM, while 1 and 3 μM had no effect.

**Figure 3:**
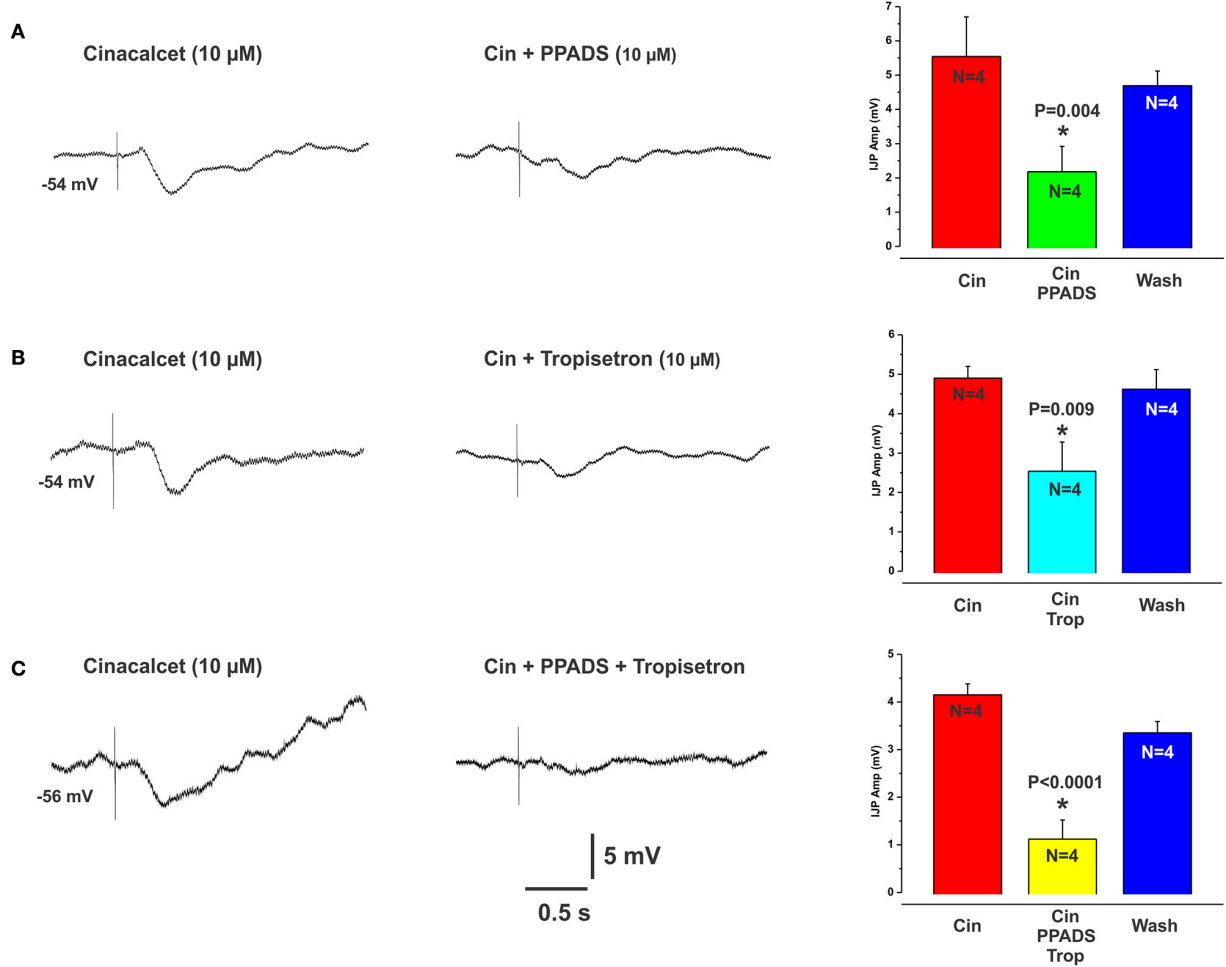
Cinacalcet evoked IJPs were reduced by either PPADS or tropisetron. **(A–C)** (left panels) show intracellular recordings of IJPs evoked by the CaSR agonist cinacalcet (10 μM). These were indistinguishable from IJPs evoked by L-Phe and shared the same pharmacological profile. PPADS (10 μM) or tropisetron (5-HT_3_ and 5-HT_4_ receptor antagonist, 10 μM) significantly reduced these IJPs by 60 and 49% respectively (**A,B** middle panels and histograms). The combination of PPADS and tropisetron was slightly more effective than either antagonist alone (74% reduction, **C** middle panel and histogram).

The CaSR antagonist NPS2143 reversibly reduced L-Phe evoked IJPs, at 10 and 30 μM concentrations by 48% (mean IJP amplitude L-Phe 4.2 ± 0.4 mV, NPS 10 μM 2.2 ± 0.7 mV, *N* = 4, *P* = 0.005) and 59% (L-Phe 3.9 ± 0.2 mV, 30 μM NPS 1.6 ± 0.6 mV, *N* = 6, *P* < 0.001) respectively (Figures 2A,C), while 1 and 3 μM had no effect (mean amp L-Phe 4.9 ± 0.4 mV, 3 μM NPS 2143 4.0 ± 0.6 mV, *N* = 4, *P* = 0.1; L-Phe 5.6 ± 0.2 mV, 1 μM NPS2143 5.5 ± 0.1 mV, *N* = 3, *P* = 0.7, Figure 2C). NPS 2143 did not affect electrically evoked IJPs at any concentration tested. Mucosal application of the CaSR agonist cinacalcet (10 or 30 μM) evoked responses indistinguishable from those seen evoked by L-Phe (amplitudes 3–10 mV, latencies 150–300 ms, Figure 3). They consisted of an IJP often followed by a depolarization triggering action potentials, and were sensitive to TTX (1 μM, *N* = 3). To test possible involvement of ATP or another purine in the transduction process, we tested the effects of the broad spectrum P2 purinoceptor antagonist, PPADS (10 μM), which reversibly depressed IJPs evoked by cinacalcet (mean IJP amplitude cinacalcet 5.5 ± 1.2 mV, PPADS 10 μM 2.2 ± 0.7 mV, *N* = 4, *P* = 0.004, Figure 3A). To determine whether mucosal serotonin has a role in sensory transduction, we examined the effect of the combined 5-HT_3_ and 5-HT_4_ antagonist tropisetron (10 μM), which depresses responses to L-Phe, and found that this antagonist reversibly reduced cinacalcet evoked IJPs (cinacalcet 4.9 ± 0.3 mV, tropisetron 10 μM 2.5 ± 0.7 mV, *N* = 4, *P* = 0.009, Figure 3B). Together PPADS and tropisetron reduced the IJPs by 74% (cinacalcet 4.2 ± 0.2 mV, PPADS and tropisetron 1.1 ± 0.4 mV, *N* = 4, *P* < 0.0001, Figure 3C). Thus, the pharmacology of responses to cinacalcet was indistinguishable from our previously published findings for the pharmacology of L-Phe evoked responses.

### L-glutamate evoked IJPs

**Figure 4:**
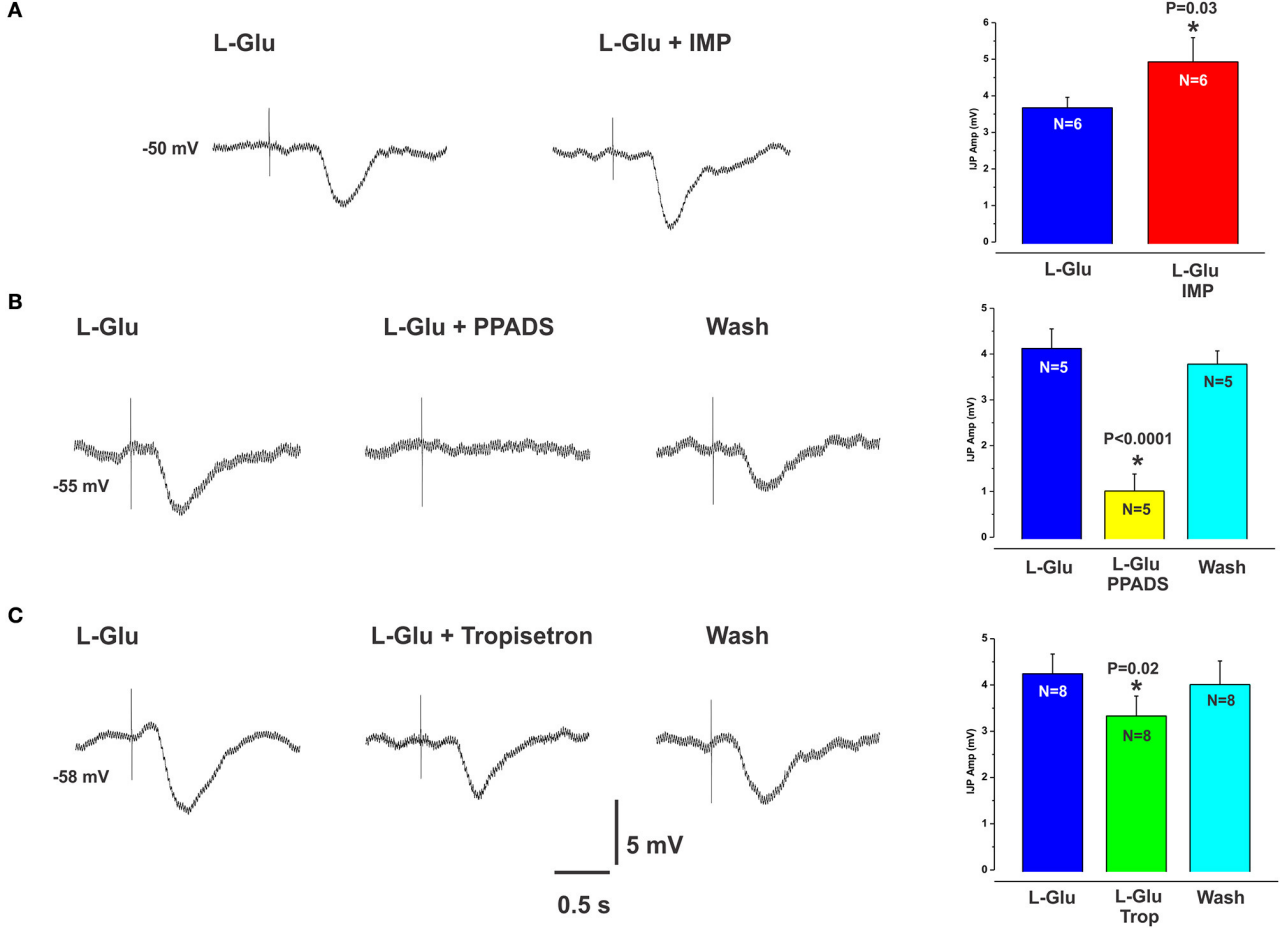
L-Glu evoked IJPs were enhanced in the presence of IMP and were more sensitive to PPADS than tropisetron. **(A)** shows an IJP evoked by spritzing L-Glu alone (left panel) and then together with IMP (right panel). The amplitudes of L-Glu evoked IJPs were significantly increased in the presence of IMP (**A**, histogram). **(B,C)** (left panels) also show IJPs evoked by L-Glu. These were reduced by PPADS (**B**, middle panel and histogram) or tropisetron (**C**, middle panel and histogram) but the effect of PPADS (75% reduction) was much larger than that of tropisetron (20% reduction).

L-glutamate (L-Glu, 30 mM), which is known to elicit the taste “umami” via activation of the T1R1/T1R3 receptor complex, evoked IJPs essentially indistinguishable from those produced by L-Phe. The activity of L-Glu at the T1R1/T1R3 receptor is enhanced in the presence of inosine monophosphate (IMP) (Zhang et al., 2008). We tested this by spritzing L-Glu or L-Glu together with IMP at the same mucosal locations. IMP (10 mM) significantly increased the amplitudes of L-Glu evoked IJPs compared with L-Glu alone (mean IJP amplitude L-Glu 3.7 ± 0.3 mV, L-Glu + IMP 4.9 ± 0.7, *P* = 0.03, *N* = 6, Figure 4A). IMP alone when applied to the mucosa evoked IJPs in 1 preparation out of 4 (latencies ranged from 150 to 270 ms, amplitudes 5–7 mV). The mean amplitude of L-Glu evoked IJPs was unchanged in the presence of nicardipine and hence responses to L-Glu do not depend on L-type calcium channels (L-Glu in nicardipine 4.2 ± 0.4 mV, N = 4).

There were no differences between L-Phe and L-Glu evoked IJPs when they were applied at the same location whether L-Phe or L-Glu was tested first (mean amp: L-Phe 3.8 ± 0.2 mV, L-Glu 4.0 ± 0.4 mV, *P* = 0.7, mean latency: L-Phe 0.20 ± 0.01 s, L-Glu 0.24 ± 0.01 s, *P* = 0.1, *N* = 4).

### Receptors involved in L-Glu evoked inhibitory reflexes

**Figure 5:**
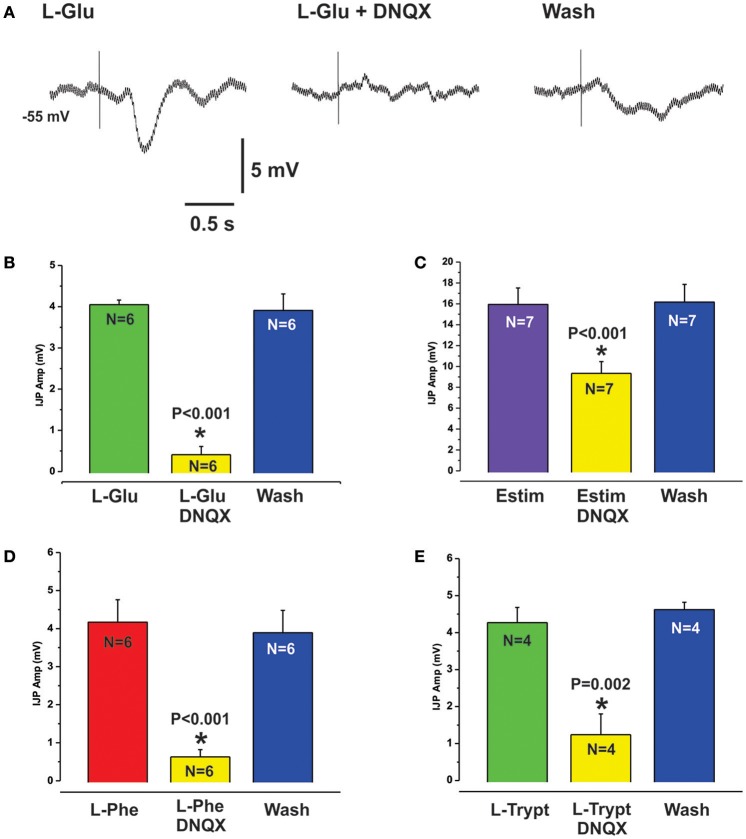
L-Glu evoked reflexes were sensitive to AMPA receptor blockade. **(A)** shows intracellular recordings of L-Glu evoked IJPs in control (left panel), in the presence of the AMPA receptor antagonist DNQX (10 μM, middle panel) and after washout (right panel). DNQX almost abolished L-Glu evoked IJPs (88% reduction, see also histogram at **B**). Electrically evoked IJPs were also significantly reduced by DNQX but to a lesser degree (41% reduction, histogram **C**). L-Phe **(D)** and L-Trypt **(E)** evoked IJPs were reduced by DNQX by 85% and 72% respectively.

L-Glu evoked IJPs were unaffected by the CaSR antagonist NPS 2143 (10 μM, *N* = 4, Figures 2B,D). However, they were reduced by PPADS (10 μM, Figure 4B) or tropisetron (5-HT_3_ and 5-HT_4_ receptor antagonist, 10 μM, Figure 4C) but the effect of PPADS (75% reduction) was much larger than that of tropisetron (20% reduction). Neither PPADS nor tropisetron affected electrically evoked IJPs. The AMPA receptor antagonists DNQX and CNQX (both 10 μM) each reversibly reduced IJPs evoked by L-glutamate by 88 and 79% respectively (Figures 5A,B). L-Phe evoked IJPs were also reduced by DNQX by 85% (L-Phe 4.2 ± 0.6 mV, DNQX 0.6 ± 0.2 mV, *N* = 6, *P* < 0.001 Figure 5D). Similar findings were obtained with the other aromatic amino acid, L-trypt (L-trypt 4.3 ± 0.4 mV, DNQX 1.2 ± 0.6 mV, *N* = 4, *P* = 0.002 Figure 5E). However, DNQX also reduced electrically stimulated IJPs by 41% (Figure 5C) and IJPs evoked when the stimulating electrode was placed directly onto the circular muscle by 24% (control 14.7 ± 0.6 mV, DNQX 11.1 ± 1.4 mV, *N* = 6, *P* = 0.04). The latter observation might be due to a non-specific action of DNQX at inhibitory neuromuscular junctions. Neither BAY367260 (mGluR5 antagonist, 10 μM) nor 2AP5 (NMDA receptor antagonist, 30 μM) affected IJPs evoked by L-Glu or electrical stimulation.

## Discussion

The results of this study demonstrate involvement of extracellular CaSRs in sensing aromatic L-amino acids in guinea pig jejunal mucosa leading to the activation of local inhibitory reflexes in the circular muscle. This process involves the release of endogenous ATP and/or 5-HT from the mucosa, which suggests CaSRs might be located on EE cells containing ATP and/or EC cells. L-Glutamate evokes similar inhibitory reflexes via a mechanism that also involves mucosal ATP and/or 5-HT release, but does not require activation of CaSRs. Our results also present evidence that AMPA receptors play a role within local reflex pathways activated by L-amino acids applied to the mucosa. There are likely to be multiple sensory mediators and receptors acting as sensory transducers for the different amino acids presented to the gastrointestinal mucosa for absorption.

### CaSRs mediate local inhibitory reflexes evoked by L-amino acids

Several results demonstrate that CaSRs mediate local inhibitory reflexes. Firstly, pharmacological characterization of the inhibitory reflexes evoked by L- and D- amino acids matches the activation profile of the CaSR. The extracellular CaSR is known to be activated by aromatic L-amino acids and is stereospecific, i.e., they are selective for the L-isomer rather than the D-isomer (Conigrave et al., 2000; Wellendorph et al., 2009). The observation that D-Phe and D-Trypt were much less effective at evoking reflexes at sites where responses to L-Phe and L-Trypt were robust supports this. Further, the non-aromatic amino acids L-Leu and L-Lys, which are much less potent activators of the CaSR than L-Phe, were largely ineffective at mucosal sites that responded to L-Phe. L-Ala, a branched chain amino acid and a moderately potent activator at CaSRs (Wellendorph et al., 2009) produced IJPs similar to L-Phe. The strongest evidence, however, comes from the finding that the CaSR antagonist NPS2143 significantly reduced the amplitudes of IJPs evoked by L-Phe, by close to 60% at the highest concentration. Furthermore, mucosal application of the CaSR agonist cinacalcet produced neurally mediated inhibitory responses indistinguishable from those evoked by L-Phe and the responses from the different compounds shared the same pharmacological profile. As we have reported previously, L-Phe evokes neurally mediated reflexes involving the release of endogenous ATP and/or 5-HT from the mucosa (Gwynne and Bornstein, 2007). This conclusion was reached because PPADS or tropisetron, at concentrations known to block P2X receptors and 5-HT_3_ and 5-HT_4_ receptors in the guinea pig respectively, reduced amino acid evoked responses but did not affect electrically evoked IJPs. Thus, the antagonists most likely act at the level of the sensory transduction process in the mucosa and not at synapses within the reflex pathway(s) activated. Cinacalcet evoked IJPs were blocked by tetrodotoxin and reduced significantly by the same concentrations of PPADS or tropisetron, also suggesting the involvement of endogenous ATP and 5-HT release. Our data suggest involvement of other sensory mediators and receptors as well since combining these two antagonists did not completely abolish amino acid or cinacalcet evoked IJPs. We have previously shown that CCK does not appear to be involved at least in the case of the amino acid evoked IJPs in the guinea pig (Gwynne and Bornstein, 2007). This contrasts recent studies in mouse cell lines which demonstrate CCK release evoked by L-Phe and activation of the CaSR (Hira et al., 2008; Liou et al., 2011; Wang et al., 2011). A possible candidate receptor molecule is the T1R1/T1R3 heterodimer, which senses umami taste elicited by L-Glu. L-Phe has been shown to cause CCK secretion from mouse STC-1 cells via T1R1/T1R3 receptors (Daly et al., 2013). The mGluR_4_ receptor has also been shown to function as a broad sensor of L-amino-acids (Nelson et al., 2002; Chaudhari et al., 2009). Both the T1R1/T1R3 receptor and mGluR4 have been found to be expressed on mucosal EE cells in various species or in EE cell lines including isolated human EC cells (Conigrave and Hampson, 2006). The identities of other sensory mediators and receptor molecules involved in generating amino acid evoked inhibitory reflexes in the guinea pig remain to be determined.

### Location of CaSRs

Previous studies have localized CaSR expression to EE cells of the human colonic mucosa (Sheinin et al., 2000), gastric G cells of the human stomach (Ray et al., 1997),v and epithelial cells of the rat intestine (Chattopadhyay et al., 1998; Cheng et al., 2002; Cheng, 2012) as well as to isolated mouse intestinal I cells (Liou et al., 2011; Wang et al., 2011). Furthermore, Liou et al. (2011) demonstrated L-Phe evoked CCK release via activation of CaSRs in mouse native intestinal I cells. Our results support the idea that CaSRs are also present in the guinea-pig mucosa and are involved in sensing aromatic L- amino-acids via the release of ATP and/or 5-HT. A likely possibility is that CaSRs are localized to EE cells containing ATP and/or 5-HT containing EC cells in the guinea pig, although we did not test this directly in the current study. The exact locations of CaSRs in the guinea-pig intestine remain to be identified.

### L-Glu evoked inhibitory reflexes do not involve activation of CaSRs

L-Glu evoked IJPs were unaffected by the CaSR antagonist NPS 2143, which reduced reflexes evoked by L-Phe. Further, L-Glu evoked responses, although significantly reduced by both PPADS (75% reduction) and tropisetron (20% reduction) were much more sensitive to the former rather than the latter, whereas these antagonists were roughly equipotent in reducing L-Phe evoked reflexes. These observations indicate that L-Glu evoked reflexes do not involve CaSRs and that the mechanisms mediating inhibitory reflexes evoked by L-Glu differ from those underlying responses evoked by the other L-amino acids tested.

L-Glu is known to elicit the taste “umami,” through the G-protein coupled T1R1/T1R3 heterodimer (Nelson et al., 2002; Zhang et al., 2008; Chaudhari et al., 2009). A recent study has shown expression of this receptor complex on enteroendocrine cells of colonic sections from human, rat, mouse, and guinea pig (Kendig et al., 2014). Furthermore, the same study reported the functionally significant observation that activation of the umami taste receptor (T1R1/T1R3) initiates the peristaltic reflex and pellet propulsion in the mouse distal colon. Our results showing that application of L-Glu to the mucosa evokes local neural inhibitory pathways is consistent with the idea that the T1R1/T1R3 taste receptor complex plays a role in sensing luminal nutrients and activating appropriate intestinal motor patterns. Another observation from our study that indicates the T1R1/T1R3 taste receptor complex might be involved in mediating L-Glu evoked responses is that L-Glu evoked IJPs were enhanced in the presence of IMP, which is consistent with previous knowledge of T1R1/T1R3 activation (Zhang et al., 2008). Overall, our data indicate L-Glu evoked reflexes do not involve activation of CaSRs, mGluR5 receptors nor NMDA receptors and are consistent with the idea that the umami taste receptor is involved, however, further experiments are required to be certain. It also remains to be seen if the umami receptor is partly involved in mediating responses evoked by L-Phe or other amino acids and which other receptor(s) might act as physiological sensors of amino acids in the guinea-pig small intestine.

### Receptors within reflex pathways activated by L-amino acids

We have previously reported that local IJPs evoked by either L-amino acids or electrical stimuli applied to the mucosa are unaffected by blockade of nicotinic, NK1, NK3, and CGRP receptors (Gwynne and Bornstein, 2007). In this study we found that L-Glu, L-Phe, and L-Trypt evoked IJPs were sensitive to blockade of AMPA receptors with DNQX which reduced their amplitudes by between 70 and 90%. Since DNQX also reduced electrically stimulated IJPs by 41% our data indicate a role for glutamate acting at AMPA receptors within local reflex pathways activated by L-amino acids. This is a significant and novel finding since many years of investigation have revealed little evidence demonstrating a physiological role for glutamate or its receptors despite their expression on several different types of enteric neurons in guinea pigs (Galligan, 1998; Kirchgessner, 2001), rats (Burns and Stephens, 1995), and mice (Seifi and Swinny, 2016). Liu et al. (1997) showed that some myenteric AH and S neurons respond to glutamate via activation of AMPA and /or NMDA receptors indicating a possible role for glutamate in synaptic transmission within the myenteric plexus but studies demonstrating a functional role for glutamate are rare. One study identified a role for AMPA receptors in modulating neurotransmitter release and peristalsis in the isolated guinea-pig colon (Giaroni et al., 2000) and a more recent study has demonstrated a role for AMPA receptors in modulating the force of spontaneous longitudinal muscle contractions in the mouse distal colon (Seifi and Swinny, 2016). Thus, our results showing that AMPA receptors might play a role in local neural reflexes excited by nutrients is an exciting addition to the relatively small body of research that has suggested functional roles for glutamate in the ENS. Other transmitters involved at synapses in these reflex pathways remain to be identified.

## Conclusions

This study identifies the extracellular CaSR as one of the receptors mediating L-amino acid evoked local inhibitory reflexes in the guinea pig jejunum. L-Glu evokes similar inhibitory reflexes via the release of mucosal ATP and/or 5-HT, but they do not involve activation of CaSRs. This study has also identified a role for glutamate acting at AMPA receptors in mediating local reflex pathways evoked by L-amino acids.

### Physiological significance

Amino acids are essential for nutrition. This study demonstrates the physiological coupling of amino acid sensing by CaSRs in the guinea pig mucosa with activation of enteric neural pathways likely to be involved in modulating intestinal motor patterns to facilitate absorption. These results contribute to the understanding of mechanisms by which the ENS detects nutrients and generates complex motor patterns such as segmentation.

## Ethics statement

This study was carried out in accordance with the recommendations and guidelines of the University of Melbourne Animal Experimentation Ethics Committee. The protocol was approved by the University of Melbourne Animal Experimentation Ethics Committee.

## Author contributions

RG assisted with experimental design, undertook experiments, analyzed data and wrote the manuscript, KL performed experiments and analyzed data, LP obtained funds, designed experiments and revised manuscript, JB obtained funds, designed experiments and revised manuscript. All authors approved the final manuscript.

### Conflict of interest statement

The authors declare that the research was conducted in the absence of any commercial or financial relationships that could be construed as a potential conflict of interest.
